# Research on lung cancer and its funding, 2004–2018

**DOI:** 10.3332/ecancer.2020.1132

**Published:** 2020-11-03

**Authors:** Mursheda Begum, Isobel Urquhart, Grant Lewison, Fouad Fouad, Richard Sullivan

**Affiliations:** 1 Queen Mary University of London, Business School, Mile End Road, London E1 4NS, UK; 2 King’s College London, Institute of Cancer Policy, Guy’s Hospital, London SE1 9RT, UK; 3 University of Bristol, School of Geographical Sciences, University Road, Bristol BS8 1SS, UK; 4 Faculty of Health Science, American University of Beirut, Beirut, Lebanon; a https://orcid.org/0000-0002-4493-1216

**Keywords:** lung cancer research, disease burden, research domains, funding

## Abstract

Although smoking is declining in high-income countries, the relative burden from its most well-known consequence, lung cancer, continues to increase, especially in low-income countries. We examined the amount, types, geographical origins and funding of research on lung cancer as revealed by papers in the Web of Science over the 15 years, 2004–2018. The annual number of lung cancer research papers increased over the study period from 2,157 to 8,202, but as a percentage of all biomedical research in Western Europe and North America they only accounted for one-eighth of the percentage of the disease burden. Lung cancer increased its share of cancer research from 4.4% to 6.5%, mainly because of the greatly expanded output from China in 2014–2018 which published almost one-third of the world’s total on a fractional count basis. For almost all other countries, their lung cancer presence in cancer research has declined over the 15 years. However, only 15% of the Chinese papers were co-authored internationally and its research was focussed on treatment rather than prevention. Support for lung cancer research is primarily from the government rather than charity. There is therefore an urgent need to increase support for lung cancer research, and for more international collaboration, especially in low-income countries where the disease burden is growing rapidly, and in neglected domains, such as screening and palliative care.

## Introduction

Lung cancer remains the most burdensome manifestation of cancer in almost all countries, as measured by disability-adjusted life years (DALYs). This indicator comprises years of life lost to the disease (compared with life expectancy in Japan, currently the country with the highest value) plus years lived with a disability of given severity (estimated by the World Health Organization, WHO) as a number between zero and unity, the most severe. Lung cancer accounted for nearly 17% of the world cancer burden in 2015 and over 1.5% of the total disease burden from all causes. (By way of comparison, breast, stomach and colorectal cancer each accounted for 8% of the total for all cancers in that year.) This percentage was 4.5% in Western Europe and 4.0% in North America, where it was beginning to decline. It was much less in Africa (0.14%) but had increased by 65% from the percentage burden in 2000. The countries with the highest percentages of all DALYs from lung cancer in 2015 were mostly in Europe (18 of the top 21), ranging from Hungary (6.3%) to Norway (4.1%). However, these were not the countries with the highest consumption of cigarettes (https://tobaccoatlas.org/topic/consumption/) because this is increasing rapidly in some countries, such as China (where over half the men smoke and two in every five cigarettes are smoked [1, 2]), and as a result, the death toll has not yet been fully manifested.

Although smoking is well-known as a main causative factor for lung cancer, an increasing number of these cancers are not so attributable [3]. The potential causes include genetics, notably the EGFR mutation [4] in Asian females. Other factors are residential radon [5], chronic obstructive pulmonary disease [6] and being underweight [7]. The effects of alcohol consumption are disputed: it seemed to have a positive effect on the disease in Spanish women [8], but a larger meta-analysis suggested that abstinence (as opposed to moderate consumption of wine or spirits) was not beneficial [9]. Second-hand smoke is certainly a causative factor [10], but will be declining as a result of the restrictions on smoking in public spaces that have been imposed in most high-income countries [11, 12]. Other measures, such as curbs on advertising, steep increases in cigarette prices and graphic warnings on packs of the health damage caused by smoking, are intended to help smokers to quit [13–22]. In combination, they have meant that in these countries the ratio between the percentages of all DALYs attributable to lung cancer in 2015 and in 2000 has only risen by 11%. This compares with a 33% worldwide increase, and a doubling in 23 countries, mostly low-income ones in Africa. In China, the percentage of all DALYs attributable to lung cancer has increased by 40% from 2.9% in 2000 to 4.1% in 2015, and is likely to increase further as young women are starting to take up smoking [23]. Therefore, there is an increasing need for research to investigate the factors that lead to lung cancer, especially among never-smokers, means of screening and diagnosis and better treatments [24, 25].

Previous studies of cancer research have all demonstrated that lung cancer is relatively neglected within the cancer research portfolio compared with its share of the burden from all cancers. The shortfall from parity is of the order of 38% in India [26], 50% in China [27] and 75% or more in ‘western’ and ‘eastern’ Europe [28, 29]. An earlier study [30] showed that lung cancer research represented about 5% of all cancer research, and that it was slowly rising as a percentage. This study builds on that one and continues the analysis to 2018. We have also investigated the sources of funding for lung cancer research.

## Methodology

### Creation of the database of lung cancer (LUNCA) papers, outputs and research types

We carried out a bibliometric analysis of research outputs worldwide during the 15 years (2004–2018) with a filter based on articles and reviews covered in the Web of Science (WoS, © Clarivate Analytics). The methodology for the selection of lung cancer papers was described previously [30]. We paid particular attention to the outputs of 25 leading countries (based on the number of papers) and determined the fractional presence of each one on all the papers in our database. For example, a paper with one French and two Chinese addresses would be categorised as FR = 0.33 and CN = 0.67. The countries are listed, with their ISO2 codes, in Table 1.

In order to put the lung cancer research outputs in context, they were compared with the outputs of biomedical research papers in the corresponding years (2014–2018) for the 25 countries, on an integer count basis. These were identified by means of another filter, based on address terms [31], which performed well in distinguishing between biomedical and non-biomedical papers in multidisciplinary journals, such as *Nature* and *Science*. The filter included the names of diseases, body parts and of places where medical research was undertaken, such as: *AIDS* or *Bethesda* or *canc* or *Daiichi* or *eye* or *family* or genet* or hepat* or *INSERM.*

The comparator was the percentage of the countries’ disease burden in 2015 attributable to lung cancer, taken from WHO data. For these leading countries, we also compared their research outputs with their wealth, as measured by their gross domestic product (GDP). We plotted the number of papers (on a fractional count basis) in 2014–2018 with their GDPs in 2015.

We employed another macro to identify those papers that were of a particular research type (or domain) by means of sets of title words and journal name strings. These are described and listed by Begum *et al* [28]. Some papers could be classed in more than one domain; others were not classed in this way.

### Analysis of the funding of lung cancer research (LUNCA)

Since 2009, the WoS has included funding information in the acknowledgement section in three searchable fields – funding organisations (FO), the grant number (FG) and full acknowledgment text (FX). However, the names of the funders are given in numerous different forms, so we gave codes to each funder so that their papers (and fractional contributions to their funding) could be identified [32]. Funding can be either explicit from the acknowledgment or implicit from the addresses. Organisations such as national labs, charity labs and commercial companies often support research in their own laboratories, so some of the addresses (in practice, about 15%) also need to be coded. We listed the funding sources for lung cancer papers for the five years, 2009–2013, and coded them. A special Visual Basic (VBA) macro then provided codes for each of the funders on each paper. Another macro then analysed the funding of each country’s papers. Not all papers had acknowledgments, either explicit or implicit. Such papers would be funded (in Europe) either by general health service funds to hospitals or general state funding of universities. We have not analysed these sources as they are typically not given as a result of a competitive peer-review process.

The main division of funding sources is into four sectors: government (central or local/regional), private non-profit (including collecting charities, endowed foundations, hospital and university own funds and other non-profits, such as professional associations and research institutes), commercial (including pharma/biotech and non-pharma) and international (e.g., the WHO and the European Union). We also identified the leading individual funders for lung cancer research in the five years, 2009–2013.

## Results

### Volume of outputs, collaboration and research domains

**Figure 1.**
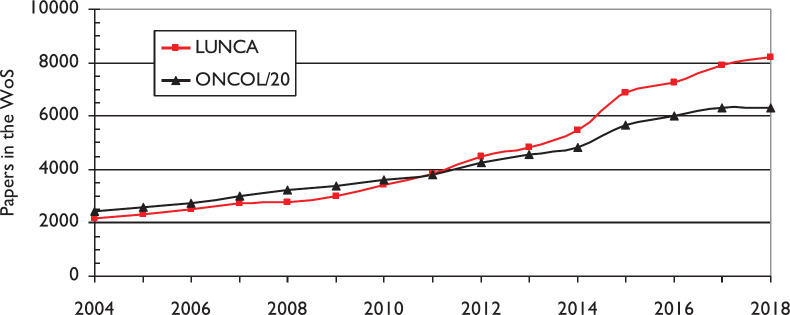
The volume of world-wide lung cancer research papers, and of all cancer research papers (divided by 20), 2004–18.

**Figure 2.**
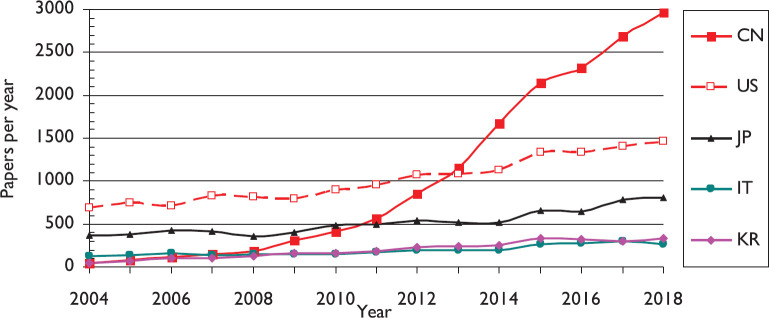
The outputs (fractional counts) of the five leading countries in lung cancer research, 2004–18. For ISO2 codes, see Table 1.

**Figure 3.**
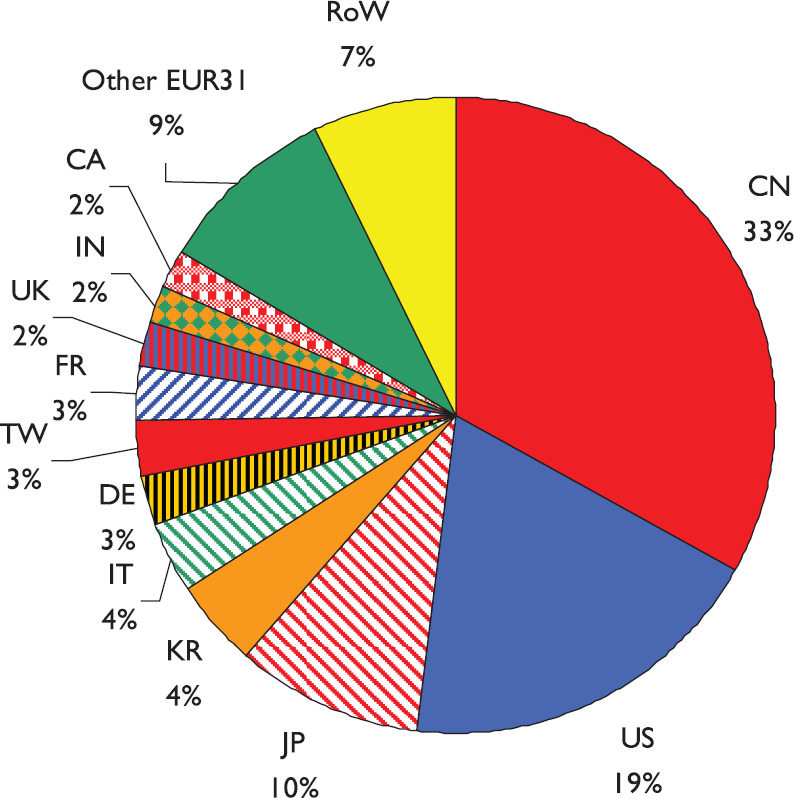
Pie diagram showing the contributions of the major countries to lung cancer research in 2014–18, fractional country counts. Country ISO2 codes are given in Table 1.

The volume of lung cancer research papers continued to expand in 2014–2018, and as a percentage of all cancer research, it actually increased from 4.4% in 2004 to 6.5% in 2018 (see Figure 1). There was a notable change in 2014–2018 from earlier years, mainly because of the much increased output from China, which grew overall from 49 papers in 2004 to 2960 in 2018 on a fractional count basis (see Figure 2). While the outputs from other countries only grew modestly, that of China expanded at a rate of about 30% per year. The result is that the Chinese output in 2014–2018 dominated the world production (see Figure 3). Together with the USA, Japan (JP) and South Korea (KR), it accounted for two-thirds of the world output in these years.

**Figure 4.**
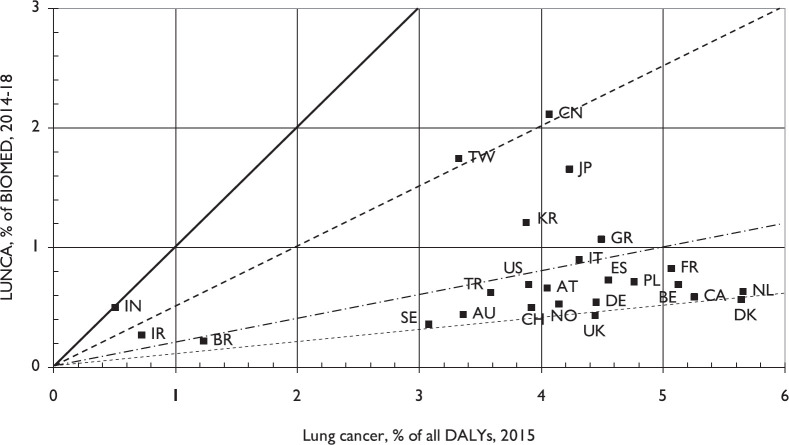
Comparison of the percentages of the leading countries’ lung cancer research (LUNCA) to their biomedical research (BIOMED) outputs in 2014-18, integer counts, with the percentages of their disease burden from lung cancer in 2015. Country ISO2 codes are given in Table 1. Lines show equivalence, half, one fifth and one tenth the corresponding percentages.

**Figure 5.**
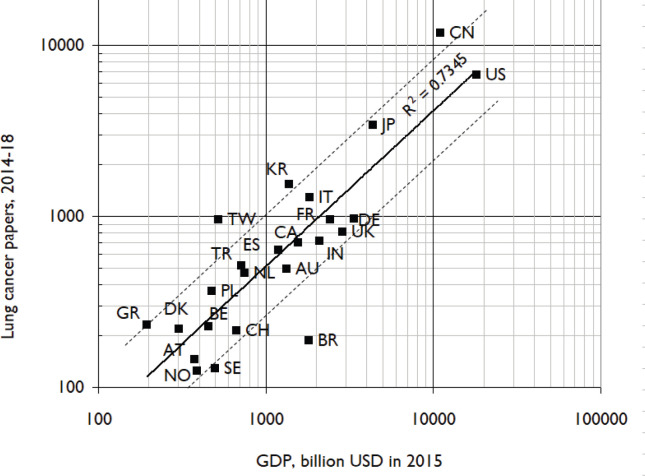
Comparison of lung cancer research outputs in 2014–18 with countries’ GDPs in 2015. Log-log scales. Country ISO2 codes are given in Table 1. Dashed lines show outputs twice and half the amounts expected on the basis of the least-squares correlation line.

Figure 4 shows the comparison of the percentages of the leading countries’ lung cancer research in 2014–2018 to their biomedical research outputs with the percentages of their disease burden attributable to lung cancer. Although India’s (IN) output is proportionate, that of China is only half that level, and most Western European countries and those in North America publish between one-fifth and one-tenth the amount that the disease burden would justify. Figure 5 shows the comparison between lung cancer research outputs in the same years with countries’ GDPs in 2015. The correlation is fairly good, with China, Japan, South Korea and Greece (GR) publishing about twice the amount expected. However, many European countries, notably Sweden (SE), Norway (NO) and Switzerland (CH) published only about half as much. Brazil (BR) published less than one-quarter of the expected amount.

**Figure 6.**
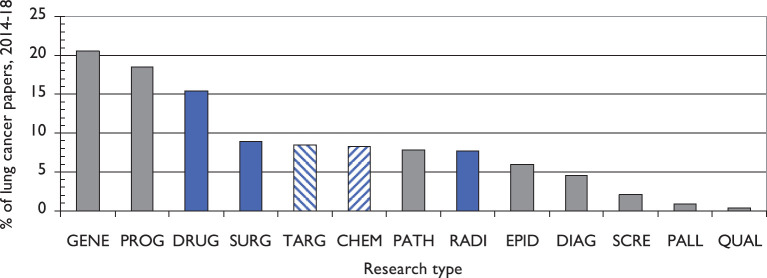
Percentages of lung cancer research papers in 2014–18 represented by 12 types of research. Note: targeted therapy (TARG) and chemotherapy (CHEM) have been combined as “DRUG”. Means of treatment shown as blue bars. GENE = genetics, PROG = prognosis, SURG = surgery, PATH = pathology, RADI =radiotherapy, EPID = epidemiology, DIAG = diagnosis, SCRE = screening, PALL = palliative care, QUAL = quality of life.

The types of research during the five years, 2014–2018, are shown in Figure 6. Genetics and prognosis are the main types of research undertaken. Drug treatment receives twice the amount of research as surgery and radiotherapy. However, there are significant variations in the relative amounts of work in the different research types or domains undertaken by the different countries (see Table 2). In order to show the countries’ relative commitment more readily, Table 2 shows this for each country, where unity indicates that the country is performing the world average amount for the stated research type or domain. Some of the cells have been tinted to show particularly high (light or bright green) or low (yellow or pink) values, so as to make the figures that depart from the mean of unity stand out. China is notably weak in screening and palliative care, research types in which the USA is strong. Japan excels in surgery, but is also weak in screening, and Italy is weak in quality of life and palliative care, research types in which the UK is strong.

The differences in specialisation suggest that more international collaboration might help to improve the outputs of some countries which appear rather weak in the above table. The amount of such collaboration for these leading countries is shown in Table 3. For China and the USA, there were 1414 collaborative papers with authors from both countries. They represented 11% of Chinese production (out of 12,730 papers) and 16% of US production (out of 8,802 papers). Of these 1,414 co-authored papers, only 19 (1.3%) were on screening, six (0.4%) on palliative care and three (0.2%) on quality-of-life; so there is clearly ample scope for more collaboration in these research domains.

### The funding of lung cancer research

**Figure 7.**
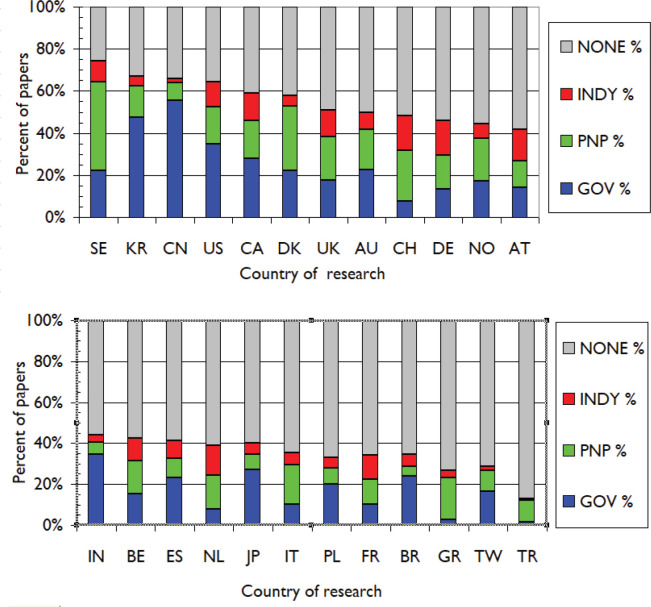
Main funding sectors (GOV = government, PNP = private-non-profit, INDY = industry) for lung cancer papers from 24 leading countries, 2009–13. For ISO2 codes, see Table 1.

Figure 7 shows the number of papers from 24 leading countries based on fractional counts that had funding from the four main sectors in 2009–2013. Of the 19,644 papers published in the five years, 11,015, or 56%, had some acknowledged explicit or implicit funding. Sweden (SE) had by far the most funded papers (75%) and Turkey (TR) had the least (13%). China (CN) and Korea (KR) showed funding for two-thirds of their papers, Japan (JP) for 40% and Taiwan (TW) for only 30%.

The major source of funds for lung cancer research was the government, with a total fractional contribution (FC) equivalent to 5711 papers out of the fractional total of 18,556 papers from these countries, or 31% of the total, coming from this sector. The private non-profit (PNP) sector provided 14% of the total. The USA depended much more on the Federal Government for research support than from charitable sources, as did most other countries, except in Western Europe. The European countries with the most support from PNP sources, relative to that from the government, were Switzerland (ratio of × 3.2), the Netherlands (×2.1), Sweden and Italy (×1.9) and Denmark (×1.4). The ratios for Turkey and Greece were both more than seven, but these results are anomalous as neither had a large PNP sector and the amount of contestable support from the government was small.

Table 4 lists the main individual sources of support from the governmental and private non-profit sectors. There is some potential separated counting: for example, some acknowledgments credit the US National Cancer Institute (NCI) and some the US National Institutes of Health (NIH). In practice, most of the support for lung cancer research from the NIH would have come from the NCI. One ‘FC’ amounts to the cost of one biomedical research paper, see below. Support from industry amounted to an average of 8% of the total, but US researchers received 40% of 1448 industrial FCs given to the 24 leading countries. However, industrial support was higher (as a fraction of total support) in Switzerland and Germany (16%), Austria and the Netherlands (14%), Canada (13%) and the UK (11%). In Table 5, the fractional contributions of the individual companies, and the groups of ones with generic codes, have all been added to give a composite industrial total for the 24 countries.

**Figure 8.**
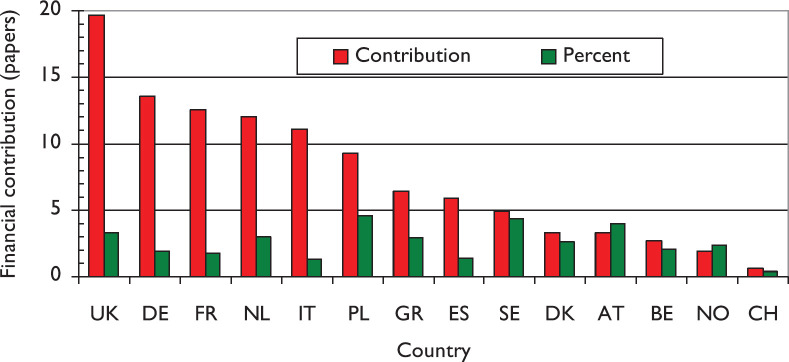
Support for lung cancer research from the European Union to 14 Member States in 2009–13.

Figure 8 shows that support from the European Union (EU) was unevenly distributed, with the UK receiving the most (20 FCs), followed by Germany (14 FCs), France (13 FCs), the Netherlands (12 FCs) and Italy (11 FCs). However, in relation to their output, Poland (4.6%), Sweden (4.4%) and Austria (4.0%) obtained the most support from the EU.

A previous study [33] that involved a survey of leading researchers in Europe to determine the costs of an individual biomedical research paper yielded an average figure (for 2013) of €255,000, but it varied with income *per caput* for three groups of countries. If we apply the formula relating average paper cost to income *per caput*, we can estimate the expenditure in the leading countries as in Table 6. The sum of the individual countries’ research expenditures is €961 million. Allowing for the estimated 241 papers missing from the total output in that year at, approximately €100,000 per paper (as most of their researchers will be from low- and medium-income countries), we should add €24 million to give an estimated total of €985 million. In 2018, with costs about 10% higher per paper, and an output of 8202 papers, the likely total research expenditure is about €1.6 billion rather than €1.0 billion.

## Discussion

It appears from Figure 4 that only India (IN) was publishing an amount of lung cancer research that was proportionate to its disease burden. China (CN) published about half the proportionate amount, but the USA and most European countries only published between one-fifth and one-tenth this amount. In view of the still increasing toll from the disease, this represents a substantial failure of most countries to tackle the problem.

As with other manifestations of cancer, there was a lack of research on palliative care for patients nearing the end of their lives. This can be a very difficult and unpleasant time as they struggle to breathe [34] and are concerned about distress to their families [35]. The only real hope, a lung transplant, remains out of reach for most of them. There may also be reluctance on the part of hospitals to treat smokers, some of whom are so addicted to their habit that they cannot give it up even when unmistakable signs of respiratory illness are present. Some countries are relatively active in this domain, such as Denmark, Poland the Netherlands and the UK, and it would be helpful if there were more international collaboration to spread best practices.

The study inevitably has some limitations. We were not able to calibrate the filter used to identify LUNCA papers, although it is likely to have high precision (over 0.9) because the ONCOL filter had a precision of 0.95. The funding analysis was carried out earlier as part of our study of European cancer papers for the European Commission [29]. Updating it to cover 2014–2018 papers would have required much time and expenditure, which were not available. Third, our analysis has mainly been restricted to high- and upper-middle income countries where most biomedical research takes place, and we have not examined the situation in Africa, for example, where lung cancer is still a minor component of the disease burden, but is increasing rapidly.

## Conclusion

There is an urgent need to increase support for lung cancer research, and for more international collaboration, especially in low-income countries where the disease burden is growing rapidly, and in neglected domains, such as screening and palliative care. As restrictions on smoking are applied increasingly, research needs to consider the other routes by which the disease is caused. There is less funding from government and industry than there is for research on other cancer anatomical sites.

## Conflict of Interest

The authors declare that they have no conflict of interest.

## Funding

This study was conducted with support of ESRC grant (ES/P010962/1) and an unrestricted grant from Roy Castle Lung Cancer Foundation, on behalf of the Global Lung Cancer Coalition of advocacy organisations. Roy Castle Lung Cancer Foundation receives unrestricted grants for the work of the Global Lung Cancer Coalition from Amgen, AstraZeneca, Boehringer Ingelheim, BMS, Lilly, Merck, Novartis, Pfizer, Roche and Takeda.

## Figures and Tables

**Table 1.** List of 25 leading countries in lung cancer research, with their ISO2 codes.

Country	ISO2	Country	ISO2	Country	ISO2	Country	ISO2
Australia	AU	France	FR	Japan	JP	Sweden	SE
Austria	AT	Germany	DE	Korea (S)	KR	Switzerland	CH
Belgium	BE	Greece	GR	Netherlands	NL	Taiwan	TW
Brazil	BR	India	IN	Norway	NO	Turkey	TR
Canada	CA	Iran	IR	Poland	PL	United Kingdom	UK
China	CN	Italy	IT	Spain	ES	United States	US
Denmark	DK						

**Table 2.** Relative commitment of the leading countries to different types (for codes, see caption to Figure 6) of lung cancer research, 2014–18. Values > 2.0 tinted bright green; values > 1.414 tinted pale green; values < 0.707 tinted pale yellow; values < 0.5 tinted pink. Fractional counts of country addresses.

	CHEM	DIAG	EPID	GENE	PALL	PATH	PROG	QUAL	RADI	SCRE	SURG	TARG
CN	1.20	0.84	0.89	1.33	0.40	1.11	1.05	0.53	0.57	0.31	0.59	0.82
US	0.74	0.90	1.25	0.78	1.76	0.85	0.92	1.68	1.50	2.57	1.03	0.99
JP	1.35	0.87	0.63	0.96	0.88	1.08	1.14	0.50	1.10	0.27	2.41	1.59
KR	0.81	0.81	0.76	1.04	1.16	1.04	1.32	0.74	0.84	0.56	1.10	0.94
IT	0.90	1.41	0.75	0.74	0.46	0.92	0.86	0.22	0.93	1.29	1.31	1.76
DE	0.78	1.44	0.84	0.75	0.78	1.37	0.93	1.01	1.60	0.85	1.12	0.93
TW	0.75	0.61	1.03	1.22	1.38	0.68	1.10	1.92	0.33	0.34	0.72	1.05
FR	1.11	1.30	1.15	0.75	0.70	0.55	0.93	0.60	1.07	0.82	1.22	1.40
UK	0.67	1.65	1.50	0.54	2.57	0.82	0.91	3.02	1.48	1.96	1.33	0.94
IN	1.10	2.50	0.86	0.85	0.42	1.27	0.51	0.20	0.53	0.20	0.35	0.69
CA	0.85	0.82	1.59	0.56	1.37	0.70	0.88	0.54	2.32	1.88	1.09	0.79
ES	0.73	1.33	1.07	0.93	0.41	1.02	1.21	0.45	0.60	1.06	0.95	1.36
TR	0.94	1.60	0.85	0.63	0.66	1.07	1.52	0.00	1.33	0.28	1.82	0.29
AU	0.64	1.42	1.23	0.64	1.43	0.89	0.84	2.24	2.03	1.33	0.86	0.67
NL	0.66	1.20	1.13	0.38	3.58	0.72	0.93	1.34	2.78	3.96	1.15	1.03
PL	0.93	1.10	0.92	1.16	3.93	1.35	0.67	8.36	0.46	1.31	1.24	0.51
GR	1.60	0.79	0.84	0.66	0.49	1.03	0.87	0.00	0.63	0.48	0.71	1.40
BE	1.03	1.11	0.40	0.67	1.04	0.60	0.60	1.25	1.66	0.69	0.73	1.21
DK	0.73	1.75	1.10	0.82	4.31	1.39	0.98	7.75	2.71	1.83	1.02	0.72
CH	1.05	0.96	0.82	0.72	0.17	0.98	0.71	1.11	1.43	1.62	1.13	1.64
BR	1.28	1.18	1.06	0.84	1.31	0.92	0.92	1.21	0.32	0.47	0.51	0.66
IR	0.83	1.10	1.28	1.23	0.00	0.76	0.72	0.00	0.99	0.73	0.39	0.28
AT	0.82	1.21	0.53	1.01	0.79	0.89	0.59	0.00	0.30	1.36	0.72	1.20
SE	0.72	1.17	1.92	0.69	0.00	1.67	1.05	0.00	1.02	0.21	0.52	0.36
NO	0.55	0.51	2.19	0.88	1.38	1.53	2.06	2.28	1.48	0.97	0.86	0.49

**Table 3.** Amount of foreign contributions to the lung cancer research papers from the leading 25 countries in 2014-18. Country ISO2 codes are given in Table 1.

ISO2	% foreign	ISO2	% foreign	ISO2	% foreign	ISO2	% foreign	ISO2	% foreign
SE	57.1	NL	45.7	DE	39.5	IT	29.2	IN	11.8
CH	54.9	CA	41.7	DK	39.1	PL	26.9	IR	11.5
BE	53.5	NO	41.1	FR	34.5	US	24.0	JP	9.8
AT	52.3	ES	40.1	GR	30.5	KR	15.2	CN	7.3
UK	46.4	AU	40.0	BR	30.5	TW	14.6	TR	6.7

**Table 4.** List of leading national public sector and private-non-profit funders of lung cancer research, 2009–13, with their nationalities and fractional contributions.

ISO	Funding organisation	FCs	ISO	Funding organisation	FCs
CN	National Natural Science Foundation	787	US	Other charities (not ACS)	97.1
US	National Cancer Institute	687	KR	Korean universities	95.0
CN	Chinese provincial governments	680	JP	Japanese universities	80.8
US	National Institutes of Health	550	TW	Taiwanese Government	74.3
JP	Ministry of Education, Science & Culture	283	IT	Assoc. Ital. Ricerca sul Cancro	73.3
US	US universities	248	CN	Other governmental funders	64.9
US	US non-profit associations	204	JP	Japanese endowed foundations	61.5
US	US endowed foundations	203	KR	Ministry of Health & Welfare	60.8
JP	Ministry of Health and Welfare	192	US	Department of Defense	58.2
KR	South Korean Government	175	US	Veterans Administration	56.8
CN	Chinese universities	155	UK	National Inst. of Health Research	49.5
KR	Ministry of Science and Technology	129	CA	Canadian Inst. for Health Res.	48.6
JP	Society for Promotion of Science	114	US	American Cancer Society (ACS)	48.6
CN	Key Program for Basic Research	101	CN	Ministry of Science & Tech’y	47.9

**Table 5.** List of leading industrial funders of lung cancer research, 2009-13, with their codes, nationalities and fractional contributions (FCs). Final digraph codes as in Table 1.

Funding code	Funding organisation	FCs	Funding code	Funding organisation	FCs
X16-IP-US	US pharma companies	170	GNH-BT-US	Genentech Inc.	25
LLL-IP-US	Eli Lilly Inc.	124	GSW-IP-UK	GlaxoSmithKline plc	23
HLR-IP-CH	Hoffman LaRoche s.a.	83	AMN-BT-US	Amgen Inc.	22
ZAT-IP-UK	AstraZeneca plc	79	X3B-BT-JP	JP biotech coys	21
PFZ-IP-US	Pfizer Inc.	74	X76-IP-FR	FR pharma coys	21
X1B-BT-US	US biotech companies	66	BMS-IP-US	Bristol Myers Squibb	21
X36-IP-JP	JP pharma companies	55	MRK-IP-US	Merck Inc. (US)	18
SLU-IP-FR	Sanofi Aventis s.a.	43	SMN-IN-DE	Siemens AG	17
NVP-IP-CH	Novartis s.a.	43	BAY-IP-DE	Bayer AG	16
X15-IN-US	US industrial companies	42	Z86-IP-KR	KR pharma coys	15
BOI-IP-DE	Boehringer Ingelheim AG	40	CHG-IP-JP	Chugai Pharma Co	14
X26-IP-DE	DE pharma companies	36	Z16-IP-CN	CN pharma coys	14
VAR-IN-US	Varian Medical Systems	27	Z1B-BT-CN	CN biotech coys	10

IN = non-pharma company; IP = pharma company; BT = biotechnology company

**Table 6.** Estimates of the expenditure on lung cancer research in 2013 in the 24 leading countries. For ISO2 codes, see Table 1.

ISO2	N	GDP/c.	CPP, €k	€m	ISO2	N	GDP/c.	CPP, €k	€ m
US	1,080	53,470	272	294	TR	98	10,970	131	13
JP	524	46,330	248	130	GR	54	22,690	170	9
CN	1,155	6,560	117	135	AU	70	65,400	311	22
IT	195	35,620	213	41	PL	49	13,240	139	7
KR	245	25,920	181	44	IN	52	1,570	100	5
DE	164	47,250	251	41	BE	22	46,340	248	5
FR	147	43,520	239	35	CH	35	90,680	395	14
UK	133	41,680	233	31	SE	24	61,710	299	7
TW	173	25,920	181	31	DK	34	61,670	299	10
CA	118	52,210	268	32	BR	33	11,690	134	4
ES	85	29,940	194	17	NO	14	102,700	435	6
NL	82	51,060	264	22	AT	20	50,390	262	5

N = number of papers (fractional counts), GDP/c. = wealth *per caput,* CPP, €k = cost per paper, €m = estimated research expenditure
